# Over 30 Years of Misidentification: A New Nothospecies *Lycoris* × *jinzheniae* (Amaryllidaceae) in Eastern China, Based on Molecular, Morphological, and Karyotypic Evidence

**DOI:** 10.3390/plants11131730

**Published:** 2022-06-29

**Authors:** Si-Yu Zhang, Ying-Feng Hu, Hao-Tian Wang, Peng-Chong Zhang, Jian-Wen Shao

**Affiliations:** 1 College of Life Sciences, Anhui Normal University, Wuhu 241000, China; zsy956042458@sina.com (S.-Y.Z.); huyf@anhu.edu.en (Y.-F.H.); WHT0607@ahnu.edu.cn (H.-T.W.); 2 Hangzhou Botanical Garden, Hangzhou 310013, China; zhang-pengchong@163.com; 3 The Key Laboratory of Conservation and Employment of Biological Resources of Anhui, Anhui Normal University, Wuhu 241000, China

**Keywords:** chloroplast genome, natural hybridization, taxonomy, nomen nudum

## Abstract

Based on the complete chloroplast genome, morphology, and karyotype evidence, we identified a new nothospecies, *Lycoris* × *jinzheniae* S.Y. Zhang, P.C. Zhang & J.W. Shao, in eastern China. This new nothospecies has been inappropriately named *Lycoris* × *albiflora* in the previous literature for more than 30 years. However, the new nothospecies resulted from the hybridization of *L. sprengeri* and *L. chinensis* and had the following characteristics: the karyotype was 2n = 19 = 3V + 16I, the leaves emerged in the spring, the ratio of filament to corolla length was approximately 1.2, tepals were slightly undulated and curved, and it was distributed throughout eastern China. These characteristics are quite different from those of *L.* × *albiflora*; thus, in this study, we named it and provided a detailed morphological description and diagnosis.

## 1. Introduction

*Lycoris* Herb. (Amaryllidaceae), first described as *L. aurea* (L’Her.) Herb., is only naturally distributed in East Asia [1]. Possessing large, beautiful, and colorful flowers, this genus of plants has great ornamental value and potential for horticultural applications. Possibly due to their highly consistent living habitats, some different species of *Lycoris* may overlap in distribution, resulting in extensive interspecific hybridization events. At present, there are 27 legitimate species names in this genus, but only 9 entities are considered original fertile diploid species, i.e., *L. chinensis*, *L. sprengeri*, *L. radiata*, *L. longituba*, *L. aurea*, *L. traubii*, *L. sanguinea*, *L. wulingensis*, and *L. tsinlingensis*. The remaining species have been proven to be or possibly to be nothospecies [1,2,3,4,5,6,7,8,9,10]. Although most F1 *Lycoris* hybrids have little ability to reproduce sexually due to disorders of their chromosome pairing during meiosis, they can vegetatively reproduce via bulb segments and can form a considerable number of clonal clusters in the wild [1,8,11,12,13,14]. Moreover, these nothospecies have a greater variety of floral colors, so they usually have higher horticultural utilization value. Therefore, accurate and legitimate naming is helpful for follow-up in-depth research and application [1,2,6,8,15].

**Figure 1:**
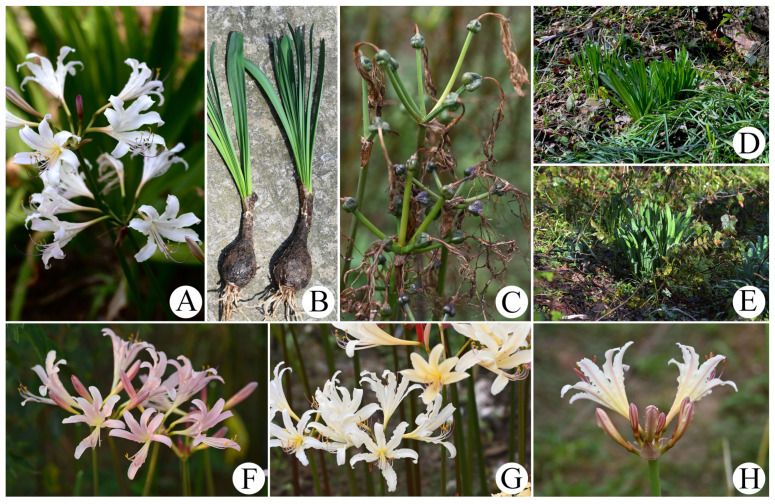
*Lycoris× jinzheniae* S.Y. Zhang, P.C. Zhang & J.W. Shao. (**A**) Inflorescence (artificial hybridization); (**B**) leaves and bulbs; (**C**) fruit (cannot bear seeds); (**D**,**E**) habitat; (**F**–**H**) variety of flower colors in the wild.

In recent years, in the process of collecting germplasm resources and performing systematic research on the genus *Lycoris*, we found that *L. sprengeri* (leaves emerging in the spring, pink-blue flowers, and endemic to China) and *L. chinensis* (leaves emerging in the spring, yellow flowers, and distributed in China and South Korea) sometimes overlap in distribution, and a special type of infertile species with white flowers and leaves emerging in the spring can be found in their overlapping populations (Figure 1). These plants were formerly recognized as *Lycoris* × *albiflora* Koidz. in Chinese taxonomic and horticultural studies, and subsequent molecular systematic studies adhered to this taxonomic opinion [4,5,16]. However, through a literature review and specimen examination, we found that *L.* × *albiflora* was named after a type collected in Japan with white tepals and leaves emerging in the autumn [1,17]. After researching the karyotype and molecular phylogeny, *L.* × *albiflora* is now considered to be the nothospecies descendant of *L. traubii* (leaves emerging in the autumn, yellow flowers, and distributed in Japan and the Taiwan Province of China) and *L. radiata* (leaves emerging in the autumn, red flowers, and widely distributed in East Asia), and is unlikely to be distributed in mainland China [1,3,11,12]. Therefore, the previous identification of such special species (with white flowers and leaves emerging in the spring) may be incorrect.

In the 1980s, Ms. Lin Jin-zhen, a pioneer in the cross-breeding of *Lycoris* in China, through artificial hybridization experiments, obtained a hybrid from *L. sprengeri*♀ and *L. chinensis*♂ that was very similar to our suspected species in flower shape and color and leaf emergence time and shape. In 1990, she and her colleagues published a nomen nudum *Lycoris* × *elegans* J.Z. Lin et Hsu, with no diagnosis and specimen type assignment [1,18]. To verify whether the suspected hybrids we found in the wild were consistent with these artificial hybrids, we introduced 30 artificial hybridization bulbs from Ms. Lin to conduct molecular systematics, karyotype, and morphological studies. Here, we confirmed that this type of white-flower *Lycoris* plant in China, obtained from the hybridization of *L. sprengeri*♀and *L. chinensis*♂, is different from *L.* × *albiflora* in the origin of hybridization. Furthermore, there were significant differences between the two hybrids in terms of leaf emergence stage, leaf shape, tepal shape, and filament length (Table 1). Therefore, these special infertile species are a new nothospecies that has not been officially described. In honor of Ms. Lin’s contribution to the hybrid breeding of Lycoris, we hereby name the nothospecies as *Lycoris* × *jinzheniae* S.Y. Zhang, P.C. Zhang & J.W. Shao and describe it.

## 2. Results

### 2.1. Molecular Phylogenetics

**Figure 2:**
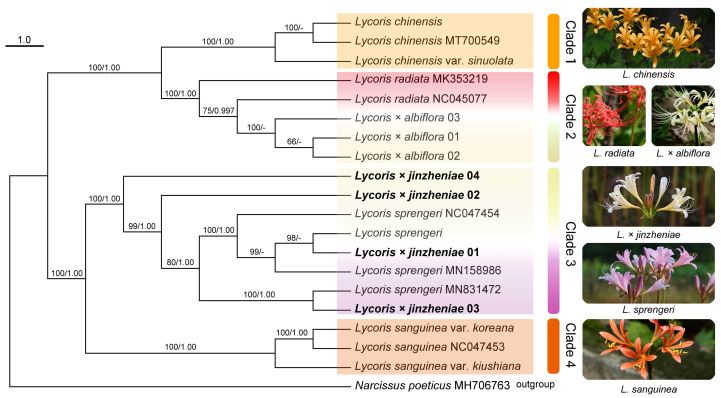
Phylogeny of *Lycoris* based on the complete chloroplast genome. Numbers above branches are maximum likelihood bootstrap values (BSs)/Bayesian posterior probability (PP). Sequences from NCBI are indicated in the figure.

Figure 2 displays the molecular phylogenetic relationship based on the complete chloroplast genome; the nineteen plants represent six species clustered into four clades, with the highest bootstrap values on the phylogenetic tree constructed by complete chloroplast genome sequences. *Lycoris chinensis* var. *sinuolata* from Korea and *L. chinensis* from China constituted Clade 1. *Lycoris radiata* from China and *L.*× *albiflora* from Japan formed Clade 2. *Lycoris sprengeri* and *L.* × *jinzheniae* (two wild plants and two artificially hybridized plants) nested together (Clade 3), and *L. sanguniea* and its two varieties clearly clustered together (Clade 4). Clade 1 and Clade 2 and Clade 3 and Clade 4 were sister groups to each other.

### 2.2. Karyotype

**Figure 3:**
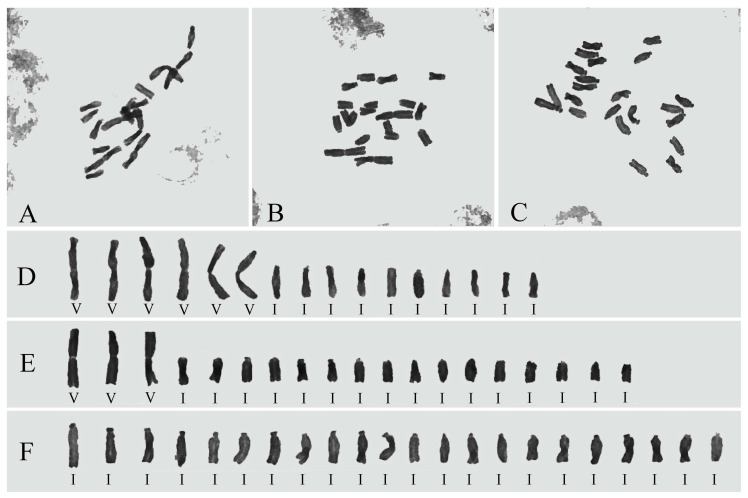
The karyotypes of *Lycoris* × *jinzheniae*, *L. chinensis*, and *L. sprengeri*. (**A**,**D**) *L. chinensis*, 2n = 6V + 10I; (**B**,**E**) *L.* *×* *jinzheniae*, 2n = 3V + 16I; (**C**,**F**) *L. sprengeri*, 2n = 22I.

We observed the karyotype of the mitotic phase of root tips of *Lycoris chinensis*, *L. sprengeri*, and *L.* × *jinzheniae* was stable, and found that *L. chinensis*’s karyotype was 2n = 16 = 6V + 10I (x = 3V + 5I), *L. sprengeri*’s karyotype was 2n = 22 = 22I (x = 11I), and *L.* × *jinzheniae*’s (including artificial hybridization) karyotype was 2n = 19 = 3V + 5I + 11I = 3V + 16I (Figure 3).

### 2.3. Morphological Characteristics and Fertility

**Figure 4:**
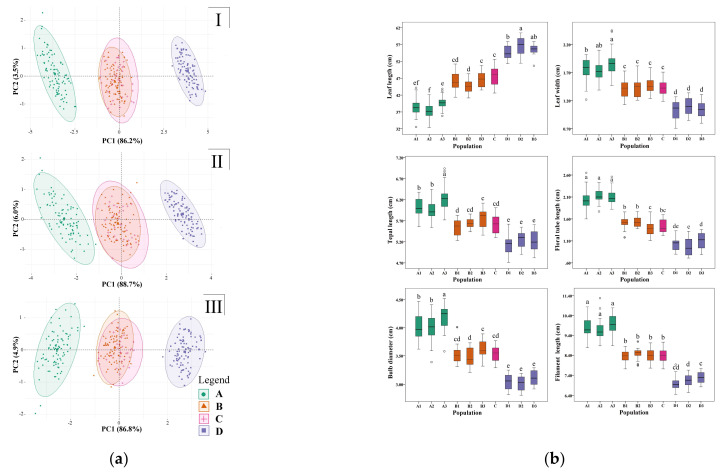
Morphological comparison of *Lycoris* × *jinzheniae*, *L. chinensis*, and *L. sprengeri* based on quantitative taxonomy: (**a**) principal component analysis diagram Ⅰ. based on all 10 morphological characters, Ⅱ. based on 5 vegetative characters, Ⅲ. based on 5 propagule characters. The letters in the legend correspond to the populations in Table 2, and the circles represent the 95% confidence intervals. (**b**) Comparison of and variation in 6 morphological characters. The codes of the abscissa correspond to the populations in Table 2. In the boxplot, the horizontal line shows the median, while the bottom and top of the box show the first and third quartiles. Boxplots marked with different letters differ significantly (post hoc test, *p* < 0.05).

Principal component analysis of five vegetative characters, five propagule characters, and their combination showed that there was obvious morphological differentiation between *L. chinensis*, *L. sprengeri* and *L.* × *jinzheniae* (including 30 artificial hybridization plants), and *L.* × *jinzheniae* morphologically fell between the first two. The wild populations and the artificial hybrid population were consistent based on the 95% confidence interval, suggesting that they are morphologically similar (Figure 4a). In six quantitative characteristics, there was no obvious difference in morphology among three wild populations and one artificially hybridized population of *L.* × *jinzheniae* (Figure 4b). However, the morphological differences between *L.* × *jinzheniae*, *L. chinensis*, and *L. sprengeri* were conspicuous and significant. In general, the morphological indicators of *L.* × *jinzheniae* were almost between the latter two species (Figure 4b).

**Figure 5:**
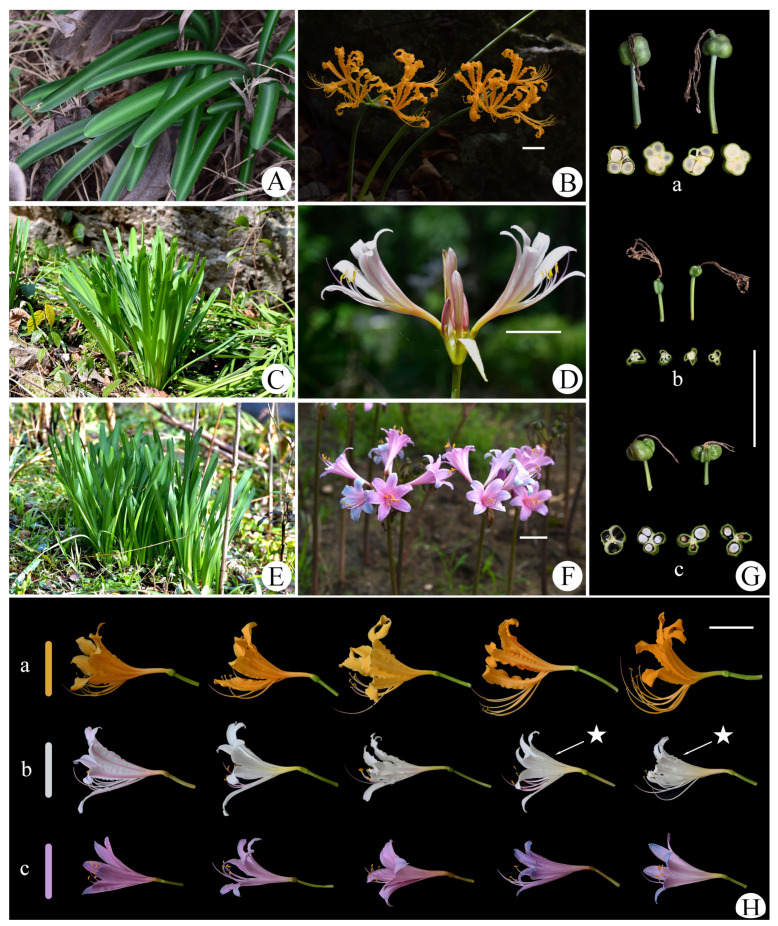
Morphological comparison of *L.* × *jinzheniae*, *L. chinensis*, and *L. sprengeri*. (**A**,**B**,**G-a**,**H-a**.) *L. chinensis*. (**C**,**D**,**G-b**,**H-b**). *L.* × *jinzheniae*. (**E**,**F**,**G-c**,**H-c**). *L. sprengeri*. (**G**). indicates the fertility difference of the three species. ★ means artificial hybridization. Scale bars = 5 cm.

To compare fertility more intuitively, we cross-sectioned the capsules of the three *Lycoris* species at the same time and found that both *L. sprengeri* and *L. chinensis* had many developing seeds, but *L.* × *jinzheniae* could not bear any filled seeds (Figure 5G). This was not an accidental phenomenon but occurred in all populations.

### 2.4. Taxonomic Treatment

*Lycoris* × *jinzheniae* S.Y. Zhang, P.C. Zhang & J.W. Shao nothosp. nov. (Figure 1):

—*Lycoris sprengeri* Comes ex Baker × *Lycoris chinensis* Traub;

—*Lycoris* × *elegans* J.Z. Lin et Hsu, nomen nudum.

Type. China, Zhejiang Province, Cixi City, Longshan Town, Dapon Mountain, 30°3′20″ N, 121°29′10″ E, 241 m, 02 Sept. 2021, *S.Y. Zhang*, *P.C.Zhang* & *K.J. Xu* ZSY210901 (holotype ANUB008508; isotype ANUB008509, ANUB008510, ANUB008511, CSH200000).

Diagnosis. *Lycoris* × *jinzheniae* is similar to *L.* × *albiflora* in the color of flowers, but it can be distinguished from the latter by leaves emerging in spring, leaf apex blunt, a ratio of filament to corolla length of 1.2:1, and slightly undulate and recurved tepal. There are also very obvious morphological differences between *L.* × *jinzheniae* and its parents, as shown in Table 1 and Figure 5.

**Table 2:** Collected information for morphological and karyotype observation of *Lycoris*.

Locality	Samples and Code	Total
China, Jiangsu Province, Wuxi City, Yixing County, Shanjuan Cave	A1: *L. chinensis* *30, B1: *L. × jinzheniae* *30, D1: *L. sprengeri* *30	300
China, Zhejiang Province, Cixi City, Longshan Town, Dapon Mountain	A2: *L. chinensis* *30, B2: *L. × jinzheniae* *30, D2: *L. sprengeri* *30	
China, Anhui Province, Chuzhou City, Langya County, Langya Mountain	A3: *L. chinensis* *30, B3: *L. × jinzheniae* *30, D3: *L. sprengeri* *30	
China, Zhejiang Province, Hangzhou City, Hangzhou Botanical Garden	C: *L. × jinzheniae* (artificial hybridization) *30	

Description. Perennial herb. Bulbs nearly oval or fusiform, 3–4 cm in diameter, and epidermis brown with fine lines. Leaves linear, often 6–10, blunt apex, 40–55 cm-long, 1.2–2 mm-wide, covered with little white powder; upper surface dark green, midvein slightly pale; bottom surface light green with a raised midrib. Inflorescence scapose, 40–60 cm-high, green or reddish-brown; two bracts, lanceolate, 3–4.5 cm-long, 1–1.5 mm-wide; flowers 5–8 per umbels, pedicels 3–5 cm-long, diameter 4–7 mm; buds rose-red; flowers usually white, sometimes pale pink or yellowish; tepals oblanceolate, 5–6.5 cm-long, approximately 8–12 mm-wide, apex slightly reversed and slightly undulated; floral tube 1–2 cm-long. Filament 7–9 cm-long, white, slightly longer than tepals, anther yellow, length of 5 mm; pistil length 7.5–8.5 cm, apex purplish-red, lower part white. Ovary 6–8 mm in diameter, spherical, and green. Capsules three-lobed, green, infertile.

Phenology. Flowering from August to early September. Leaves emerge in mid-February and wither in late May.

**Figure 6:**
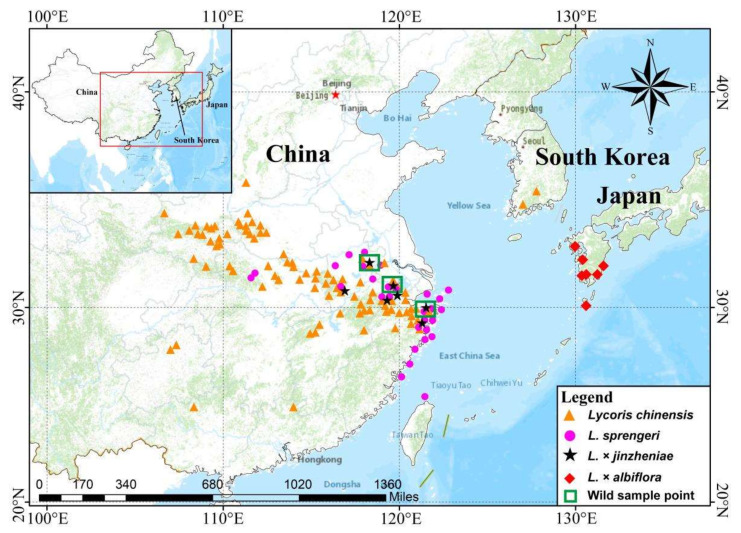
Geographical distribution of *Lycoris* × *jinzheniae* and its related species. Data based on fieldwork and references records.

Distribution and habitat. *Lycoris* × *jinzheniae* is currently found in the Anhui, Jiangsu, and Zhejiang provinces of China and grows in moist hillside forests (Figure 6). It always grows in the overlapping distribution area of *L. chinensis* and *L. sprengeri*. Occasionally, residents living around its range dig and cultivate them near their homes as horticultural plants.

Vernacular name. 秀丽石蒜 (xiù lì shí suàn).

Reproduction. It can only reproduce asexually by bulb splitting. Typically, one mature bulb can turn into three mature bulbs after 2 years.

Conservation status. Although the number of *Lycoris* × *jinzheniae* in the wild is very small, as an F1 hybrid that cannot reproduce sexually, it has no protective significance, and a large number of individual plants can be obtained through artificial hybridization. We propose classifying its conservation status as least concern (LC) according to the IUCN Red List criteria [19].

## 3. Discussion

In angiosperms, the chloroplast genome is normally maternally inherited [1,4,5]. In the phylogenetic tree that we constructed based on the chloroplast genome, *Lycoris*× *albiflora* clustered with *L. radiata* and constituted Clade 2, with a robust bootstrap (BS = 100, PP = 1.00, Figure 3), which is consistent with previous results [3]. This supports the previous viewpoint that *L. radiata* is the maternal parent of *L.* × *albiflora*, which was concluded from morphological characteristics and distribution patterns [1]. However, the wild plants of *L.* × *jinzheniae* were nested with the offspring produced by artificial hybridization between *L. sprengeri* (female parent) and *L. chinensis* (male parent), forming Clade 3 together with *L. sprengeri*. This was a sister group with *L. sanguinea* (Clade 4) (Figure 2), suggesting that the new hybrid was relatively distant from *L.* × *albiflora* in phylogenetic relationship and that *L. sprengeri* is the possible maternal parent of *L.* × *jinzheniae*.

Through the observation of multiple populations and individuals, we found that the karyotype of the new hybrid (including wild populations and artificial hybrid populations) was an odd 19 and was stable (2n = 19 = 3V + 16I), which is quite different from that of *L.* × *albiflora* (2n = 17/18 = 5V + 12I/6V + 12I) [1,12]. In the field, *L.* × *jinzheniae* concomitantly occur with *L. sprengeri* and *L. chinensis*, especially when the populations of these two species are less than 100 m apart; they can always be found within or near the *L. sprengeri* populations. Therefore, combining the distribution, karyotype, and molecular evidence, we can deduce that *L. sprengeri* (2n = 22 = 22I) is the maternal parent of the new nothospecies, and the anther accompanying plant *L. chinensis* (2n = 16 = 6V + 10I) is its male parent. These two wild diploid parents each provide a set of chromosomes that can constitute the karyotype of the new hybrid. We also note that the karyotypes of *L. longituba* and *L. aurea* are (or can be) 2n = 6V + 10I [1,11,12]. The karyotype of *L. aurea* is highly variable, and the 6V+10I type is only found in Southwest China and Hainan Province [1]. The wild population of *L. longituba* is scarce and is now only distributed in the hills along the Yangtze River in parts of the Jiangsu and Anhui provinces. Thus, we have not found that *L. longituba* or *L. aurea* are distributed near or overlapped with *L. sprengeri* thus far, which means it is very unlikely that these two species participated in the hybridization of *L.* × *jinzheniae*.

Interestingly, all the evidence we found thus far indicates that *L.* × *jinzheniae* is produced by *L. sprengeri*♀ and *L. chinensis*♂, and this hybridization occurs easily, while reverse hybridization (i.e., *L. chinensis*♀and *L. sprengeri*♂) is unlikely to occur. For example, in the artificial hybridization experiment performed in *Lycoris* by Lin et al. [18], the success rate of the hybrid combinations *L. sprengeri*♀ and *L. chinensis*♂was relatively high (approximately 40%), and a *L.* × *jinzheniae* germplasm nursery was successfully cultivated. Although some seeds were obtained by reverse hybridization, subsequent descriptions did not mention that these seeds can successfully grow seedlings. Under natural conditions, we found overlapping areas (nearly less than 100 m) of both *L. chinensis* and *L. sprengeri* in seven areas (Figure 6). *L.* × *jinzheniae* appeared in every such area, ranging from several clusters to more than 50 clusters, and they were all, without exception, closer to or on the edge of *L. sprengeri* populations. No individual plants closer to populations of *L. chinensis* have been found thus far. Furthermore, some other studies have shown that *L. sprengeri*, as a female parent, participates and forms several hybrid offsprings, but a hybrid formed from a male *L. sprengeri* parent has not yet been reported [1,4,5,20]. At present, there are similar reports of unidirectional hybridization in Ligularia, Sonneratia, Rhizophora, and other taxa, and the asymmetry of bidirectional hybridization is also prevalent in angiosperms [21,22,23]. Research suggests that parental rarity in the wild or postmating isolation may be some of the factors contributing to hybrid asymmetry [22]. The specific hybridization participation mechanism by which *L. sprengeri* can only be used as a female parent but not as a male parent is still unclear.

Morphologically, the 30 individual plants from artificial hybridization and the 90 individual plants from three wild populations were the same in terms of vegetative and propagule characteristics (Figure 5 and Figure 6). This suggests that their morphological characteristics were relatively similar and consistent, although *L.* *× jinzheniae* can be produced by multiple independent crosses (including artificial crosses). However, this new nothospecies can be distinguished from *L.*× *albiflora* by leaf emerging time (spring vs. autumn), leaf apex shape (blunt vs. acuminate), ratio of filament to corolla length (1.2:1 vs. 2:1), and tepal undulation and recurve degree (slight vs. strong).

From the above, *L.*× *jinzheniae* was quite different from *L.*× *albiflora* in origin, morphology, karyotype, and natural distribution (Figure 2, Figure 3, Figure 4, Figure 5 and Figure 6, Table 1). Here, we named it and provided a detailed morphological description and diagnosis.

## 4. Materials and Methods

### 4.1. Plant Material

From 2016 to 2021, materials were collected in the Eastern China field and Hangzhou Botanical Garden (Table 2 and Figure 6). These bulbs were cultivated in a homogenous garden located in Fengyang County, Chuzhou City, Anhui Province, for morphological and karyotype observation.

Based on pre-experiments and previous studies, we selected *L.* × *jinzheniae* and its putative parents (*L. sprengeri* and *L. chinensis*), *L.* × *albiflora* and its putative parents, *L. radiata*, and *L. sanguinea* (infertile original diploid and the sister groups of *L. sprengeri*) to construct a phylogenetic tree. Information on the newly generated complete chloroplast genome of *Lycoris* is shown in Table 3. The chloroplast genome sequences of some other species were downloaded from NCBI (Figure 2).

### 4.2. Chloroplast Genome Acquisition and Phylogenetic Analysis

Sequencing samples were obtained from leaves, which were dried in silica gel and collected from wild populations or homogenous garden. DNA extraction used a modified cetyltrime thylammonium bromide (CTAB) extraction protocol [24] with Qubit TM dsDNA HS Assay Kit (Invitrogen, Waltham, MA, USA)-mediated cleaning [25]. After polymerase chain reaction (PCR) and agarose gel electrophoresis, DNA quality control was ensured using a NanoDrop 1000 Spectrophotometer. Next-generation sequencing (NGS), which used Illumina HiSeq 6000 and FastQC, was outsourced to The Germplasm Bank of Wild Species in Southwest China (China, Kunming). We employed Getorganelle v1.7.1 and 3G raw data from the previous step to complete the assembly of the chloroplast genome [26]. The assembled chloroplast genome was annotated by PGA [27]. This study was performed on the 12 newly reported complete chloroplast genomes and 7 complete chloroplast genomes from NCBI. *Narcissus poeticus* was selected as the outgroup (Table 2, Figure 2) [20,28,29,30,31].

The sequences of 20 chloroplast genes shared by all plastomes were aligned using MACSE v2 in PhyloSuite [32,33]. The phylogenetic relationship, which included maximum likelihood (ML) and Bayesian inference (BI) methods, was implemented in IQtree, and MrBayes with the best-fit model of DNA substitution estimated by ModelFinder [34,35,36]. ML analysis was conducted using the GTR+G+I model with 1000 bootstrap replicates, and Bayesian analysis was constructed using MrBayes with 8 independent chains for 1,000,000 generations and sampling every 1000 generations [37].

### 4.3. Karyotype Observation

The actively dividing root tips were obtained by burying the bulb in moist sand. Root tips were treated with 0.1% colchicine for 12 h and then treated with fixative solution (glacial acetic acid: absolute ethanol ratio = 1:3) for 24 h in the dark. The samples were washed with water, soaked in 1 mol/L hydrochloric acid, and placed in a water bath at 60 °C for 15 min [8,10,11,12,38]. After washing with water, the root tips were stained with modified phenol fuchsin for 15 min, and the karyotypes were observed by pressing temporary mounts. Twenty bulbs of each species in each population were selected to verify the accuracy, photographed with an electronic eyepiece, and processed using Photoshop.

Some scholars divide the chromosomes of *Lycoris* into three categories based on shape, M, T, and A, but based on our karyotype observation findings, the A-type and T-type chromosomes are indistinguishable, and there is also morphological variation or centering between them [11,12,19,39]. Therefore, we only distinguish between V-shaped (meta or submetacentric) and I-shaped (acrocentric) karyotypes [19,39].

### 4.4. Morphological Statistical Analysis

Based on the materials in Table 1, 5 vegetative characters (leaf length, leaf width, bulb diameter, bulb weight, and leaf twist angle) and 5 propagule characters (tepal length, floral tube length, filament length, symmetry degree of flower, and undulate degree of tepal) of the species were used as units (artificial hybridization of *Lycoris* × *jinzheniae* is listed separately). SPSS ver. 19.0 was used to standardize and extract the principal components and test the cumulative contribution rate of the first two principal components. Then, the devtools and ggbiplot software packages of R v3.6.0 were used. Principal component analysis was performed on all traits, vegetative traits, and propagule traits [37].

Three vegetative characters (leaf length, leaf width, and bulb diameter) and three propagule characters (tepal length, floral tube length, and filament length) were selected, and SPSS ver. 19.0 was used to make boxplots to compare morphological differences and test for significance [10].

## Figures and Tables

**Table 1:** Collected information for morphological and karyotype observation of *Lycoris*.

Characters	*L. × jinzheniae*	*L. chinensis*	*L. sprengeri*	*L*. *× albiflora*
Emerging period of leaf	Spring	Spring	Spring	Autumn
Degree of leaf twist	Slight	No	Strong	No
Corolla color	White, sometimes pale pink or yellowish	Yellow	Pink-blue	Milky white or yellowish
Degree of tepal undulation	Slight	Strong	No	Strong
Degree of tepal recurve	Slight	Strong	Slight	Strong
Type of flower symmetry	Central or bilateral symmetry	Bilateral symmetry	Centrosymmetric	Bilateral symmetry
Approximate ratio of filament to corolla length	1.2:1	1.3:1	1.2:1	2:1
Fertility	Infertile	Fertile	Fertile	Infertile
Karyotype	2n = 3V + 16I	2n = 6V + 10I	2n = 22I	2n = 5V + 12I/6V + 12I
Distribution	Eastern China	China and South Korea	Eastern and Central China	Japan

**Table 3:** Information for newly generated complete chloroplast genome of Lycoris.

Individual	Locality	GenBank acc. no
*L. × jinzheniae* 1	China, Jiangsu Province, Wuxi City, Yixing County, Shanjuan Cave	ON611628
*L. × jinzheniae* 2	China, Zhejiang Province, Cixi City, Longshan Town, Dapeng Mountain	ON611629
*L. × jinzheniae* 3	★China, Zhejiang Province, Hangzhou City, Hangzhou Botanical Garden	ON611630
*L. × jinzheniae* 4	★China, Zhejiang Province, Hangzhou City, Hangzhou Botanical Garden	ON611631
*L. sprengeri*	China, Jiangsu Province, Wuxi City, Yixing County, Shanjuan Cave	ON611639
*L. sanguinea* var. *kiushinan*	Japan, Kyushu Island, Kagoshima Prefecture, Kirishima City	ON611637
*L. sanguinea* var. *koreana*	South Korea, Jeju Special Self-Governing Province, Hallim-eup	ON611638
*L. chinensis*	China, Jiangsu Province, Wuxi City, Yixing County, Shanjuan Cave	ON611636
*L. chinensis* var. *sinulata*	South Korea, Jeollanam-do, Gangjin-gun	ON611635
*L. × albiflora* 1	Japan, Kyushu Region, Kagoshima Prefecture, Kirishima City	ON611632
*L. × albiflora* 2	Japan, Kyushu Region, Kagoshima Prefecture, Amami City	ON611633
*L. × albiflora* 3	Japan, Honshu Region, Hyogo Prefecture, Akashi City	ON611634

(★: artificial hybridization).

## Data Availability

The molecular data that support the findings of this study are openly available in GenBank (see Table 3).

## References

[1] Hsu P.S., Kurita S., Yu Z.Z., Lin J.Z. (1994). Synopsis of the genus *Lycoris*. SIDA Contrib. Bot..

[2] Kim M.Y. (2004). A taxonomic review of Korean *Lycoris* (Amaryllidaceae). Korean J. Plant Taxon..

[3] Hori T.A., Hayashi A., Sasanuma T., Kurita S. (2006). Genetic variations in the chloroplast genome and phylogenetic clustering of *Lycoris* species. Genes Genet. Syst..

[4] Shi S.D., Qiu Y.X., Li E.X., Wu L., Fu C.X. (2006). Phylogenetic Relationships and Possible Hybrid Origin of *Lycoris* Species (Amaryllidaceae) Revealed by ITS Sequences. Biochem. Genet..

[5] Shi S.D., Qiu Y.X., Wu L., Fu C.X. (2006). Interspecific relationships of *Lycoris* (Amaryllidaceae) inferred from inter-simple sequence repeat data. Sci. Hortic..

[6] Yoo Y.K., Yuan T., Lee J.S., Lee A.K., Roh M.S., Kurita S., Suh J.K. (2011). Species relationships of *Lycoris* endemic to Korea evaluated by RAPD and SNPs of nrDNA-ITS regions. Hortic. Environ. Biotechnol..

[7] Quan M.H., Ou L.J., She C.W. (2013). A New Species of *Lycoris* (Amaryllidaceae) from Hunan, China. Novon.

[8] Meng W.Q., Zheng L., Shao J.W., Zhou S.B., Liu K. (2018). A new natural allotriploid, *Lycoris* × *hubeiensis* hybr. nov. (Amaryllidaceae), identified by morphological, karyological andmolecular data. Nord. J. Bot..

[9] Lu J.Y., Wang T., Wang Y.C., Zhang P.C. (2020). *Lycoris tsinlingensis* (Amaryllidaceae), a new species from shaanxi, China. Ann. Bot. Fenn..

[10] Zhang S.Y., Huang Y., Zhang P., Chen Y.B., Shao J.W. (2021). *Lycoris wulingensis*, a dwarf new species of Amaryllidaceae from Hunan, China. PhytoKeys.

[11] Kurita S. (1986). Variation and Evolution in the Karyotype of *Lycoris*, Amaryllidaceae I. General Karyomorphological Characteristics of the Genus. Cytologia.

[12] Kurita S. (1987). Variation and evolution on the karyotype of *Lycoris*, Amaryllidaceae II. Karyotype analysis of ten taxa among which seven are native to China. Cytologia.

[13] Rieseberg L.H. (1997). Hybrid origins of plant species. Annu. Rev. Ecol. Evol. Syst..

[14] Schumer M., Rosenthal G.G., Andolfatto P. (2014). How common is homoploid hybrid speciation?. Evolution.

[15] Turland N.J., Wiersema J.H., Barrie F.R., Greuter W., Hawksworth D.L., Herendeen P.S., Knapp S., Kusber W.H., Li D.Z., Marhold K. (2018). Names of Hybirds. International Code of Nomenclature for Algae, Fungi, and Plants (Shenzhen Code), Proceedings of the Nineteenth International Botanical Congress, Shenzhen, China, 23–29 July 2017.

[16] Xu Y., Hu B.Z., Huang X.L., Fan G.J., Pei J., Ding Z.Z. (1985). Amaryllidaceae. Flora Reipublicae Popularis Sinicae.

[17] Koidzumi G. (1924). Contributiones ad Cognitionem Floræ Asiæ Orientalis (continued from Vol. XXXVII p. 59). Bot. Mag..

[18] Lin J.Z., Yu Z.Z., Xu B.S. Hybridization and breeding of *Lycoris*. Proceedings of the International Symposium Botanical.

[19] IUCN (2022). Guidelines for Using the IUCN Red List Categories and Criteria. Version 15. Prepared by the Standards and Petitions Committee. https://www.iucnredlist.org/resources/redlistguidelines.

[20] Liu K., Sun L., Meng W.Q., Zhu H., Zhang D., Wang J.X. (2022). Comparative genomics and phylogenetic perspectives of sixfertile *Lycoris* species endemic to East Asia based on plastome characterization. Nord. J. Bot..

[21] Tiffin P., Olson S., Moyle L.C. (2001). Asymmetrical crossing barriers in angiosperms. Proc. R. Soc. Lond. B.

[22] Zhou R.C., Gong X., Boufford D., Wu C.I., Shi S.H. (2008). Testing a hypothesis of unidirectional hybridization in plants: Observations on Sonneratia, Bruguiera and Ligularia. BMC Evol. Biol..

[23] Ma Y.P., Xie W.J., Tian X.L., Sun W.B., Wu Z.K., Milne R. (2014). Unidirectional hybridization and reproductive barriers between two heterostylous primrose species in north-west Yunnan, China. Ann. Bot..

[24] Doyle J.J., Doyle J.L. (1987). A rapid DNA isolation procedure for small quantities of fresh leaf tissue. Phytochem. Bull..

[25] Larridon I., Walter H.E., Guerrero P.C., Duarte M., Cisternas M.A., Hernández C.P., Bauters K., Asselman P., Goetghebeur P., Samain M. (2015). An integrative approach to understanding the evolution and diversity of Copiapoa (Cactaceae), a threatened endemic Chilean genus from the Atacama Desert. Am. J. Bot..

[26] Jin J.J., Yu W.B., Yang J.B., Song Y., Claude W., Yi T.S., Li D.Z. (2020). GetOrganelle: A fast and versatile toolkit for accurate de novo assembly of organelle genomes. Genome Biol..

[27] Qu X.J., Moore M.J., Li D.Z., Yi T.S. (2019). PGA: A software package for rapid, accurate, and flexible batch annotation of plastomes. Plant Methods.

[28] Könyves K., Bilsborrow J., David J., Culham A. (2018). The complete chloroplast genome of *Narcissus poeticus* L. (Amaryllidaceae: Amaryllidoideae). Mitochondrial DNA Part B.

[29] Zhang F.J., Shu X.C., Wang T., Zhuang W.B., Wang Z. (2019). The complete chloroplast genome sequence of *Lycoris radiata*. Mitochondrial DNA Part B.

[30] Zhang F.J., Zhuang W.B., Shu X.C., Wang T., Wang Z. (2019). Complete chloroplast genome of *Lycoris sprengeri* (Amaryllidaceae) and genetic comparison. Mitochondrial DNA Part B.

[31] Zhang F.J., Wang T., Shu X.C., Wang N., Zhuang W.B., Wang Z. (2020). Complete Chloroplast Genomes and Comparative Analyses of *L. chinensis*, *L. anhuiensis*, and *L. aurea* (Amaryllidaceae). Int. J. Mol. Sci..

[32] Ranwez V., Douzery E.J.P., Cambon C., Chantret N., Delsuc F. (2018). MACSE v2: Toolkit for the Alignment of Coding Sequences Accounting for Frameshifts and Stop Codons. Mol. Biol. Evol..

[33] Zhang D., Gao F.L., Jakovlić I., Zou H., Zhang J., Li W.X., Wang G.T. (2020). PhyloSuite: An integrated and scalable desktop platform for streamlined molecular sequence data management and evolutionary phylogenetics studies. Mol. Ecol. Resour..

[34] Minh B.Q., Nguyen M.A., von Haeseler A. (2013). Ultrafast approximation for phylogenetic bootstrap. Mol. Biol. Evol..

[35] Nguyen L.T., Schmidt H.A., von Haeseler A., Minh B.Q. (2015). IQ-TREE: A fast and effective stochastic algorithm for estimating maximum-likelihood phylogenies. Mol. Biol. Evol..

[36] Kalyaanamoorthy S., Minh B.Q., Wong T.K.F., von Haeseleret A., Jermiin L.S. (2017). ModelFinder: Fast model selection for accurate phylogenetic estimates. Nat. Methods.

[37] He X., Cao J.J., Zhang W., Li Y.Q., Zhang C., Li X.H., Xia G.H., Shao J.W. (2021). Integrative taxonomy of herbaceous plants with narrow fragmented distributions: A case study on Primula merrilliana species complex. J. Syst. Evol..

[38] Chen Y.H., Li M.X. (1985). Karytype Analyses of four species (varieties) of *Lycoris* Herb. Acta Hortic. Sin..

[39] Levan L., Fredga K., Sandberg A.A. (1964). Nomenclature for centromeric position on chromosomes. Hereditas.

